# History of Tree Growth Declines Recorded in Old Trees at Two Sacred Sites in Northern China

**DOI:** 10.3389/fpls.2017.01779

**Published:** 2017-11-06

**Authors:** Yan Li, Qi-Bin Zhang

**Affiliations:** ^1^State Key Laboratory of Vegetation and Environmental Change, Institute of Botany, Chinese Academy of Sciences, Beijing, China; ^2^College of Resources and Environment, University of the Chinese Academy of Sciences, Beijing, China

**Keywords:** dendroecology, tree growth decline, sacred sites, trees, protected areas, climate change

## Abstract

Old forests are an important component in sacred sites, yet they are at risk of growth decline from ongoing global warming and increased human activities. Growth decline, characterized by chronic loss of tree vigor, is not a recent phenomenon. Knowledge of past occurrence of declines is useful for preparing conservation plans because it helps understand if present day forests are outside the natural range of variation in tree health. We report a dendroecological study of growth decline events in the past two centuries at two sacred sites, Hengshan and Wutaishan, in Shanxi province of northern China. Tree rings collected at both sites show distinct periods of declining growth evident as narrow rings. These occurred in the 1830s in both sites, in the 1920s in Wutaishan and in the 2000s in Hengshan. By comparing the pattern of grow declines at the two sites, we hypothesize that resistance of tree growth to external disturbances is forest size dependent, and increased human activity might be a factor additional to climatic droughts in causing the recent strong growth decline at Hengshan Park. Despite these past declines, the forests at both sites have high resilience to disturbances as evidenced by the ability of trees to recover their growth rates to levels comparable to the pre-decline period. Managers should consider reducing fragmentation and restoring natural habitat of old forests, especially in areas on dry sites.

## Introduction

Old forests are generally located in places where human intervention is low, such as remote areas, forest reserves or sacred sites (i.e., sites usually containing natural relics of spiritual significance) and are important for maintaining biodiversity, ecosystem functions and services (Salick et al., 2007; Gao et al., 2013; Malgorzata and Grzegorz, 2013). Declines in these forests have been documented in recent decades in many ecosystems worldwide (Laurance et al., 2000; Gibbons et al., 2008; Allen et al., 2010; Lindenmayer et al., 2012). Declines in forests are characterized by reduced tree vigor, which results in slow growth rates, dieback and mortality and are often caused by multiple agents (Amoroso et al., 2012). While the conservation of old forests in general has received increasing attention (Foster et al., 1996; Dudley et al., 2009; Lindenmayer et al., 2014), relatively few studies have investigated the history of declines of old trees in sacred sites, partly due to limited access and strict protection of these trees.

The protection of old trees in sacred sites is one of the earliest conservation measures in human society (Dudley et al., 2005, 2009). In these sites, old trees are considered to be part of the sacred entity and, thus, are spared from logging. Many sacred sites have now been incorporated into park management systems and are open to tourists. Alteration of habitat associated with park establishment activities (e.g., road access and soil compaction) and increased number of visitors, may have a negative influence on the growth of trees in these sites (Hauru et al., 2012; Sikorski et al., 2013). In addition, trees in sacred sites are also subject to global climate change and natural disturbances, and may suffer growth decline if the environmental stressors exceed a threshold of tolerance. The vulnerability of sacred sites to human activities and climate change is a major concern for the sustainable management of these sites.

Two hypotheses have been proposed to explain forest decline in sacred sites. Because forests in these areas are under strict protection, one hypothesis considers climate change and natural disturbances as the major factor affecting the growth of the old trees in these sites (Gao et al., 2013; Luo and Chen, 2015; Wong and Daniels, 2016). Others consider human activity to be the dominant factor affecting tree health in these areas because the number of visitors to the parks has increased dramatically in recent decades (Hauru et al., 2012; Sikorski et al., 2013; Reyer et al., 2015). In reality, it is difficult to attribute growth decline to either of these hypotheses because we lack comparative information on the long-term growth trajectory of trees in these sites.

The objective of this study was to identify growth decline events in the past two centuries at two sacred forests in Shanxi province of northern China and to make inferences regarding the possible causes of these declines. We used dendroecological techniques and worked at the tree level, comparing the patterns of radial growth trajectories among individual trees, rather than using the traditional approach, which is based on examining the overall growth trends of the average of all trees at a site. If a recent decline is a recurrence of past declines and falls within the historic range of variability in terms of severity, we attribute the decline to climate change. The reason for this is that parks are relatively recent and, therefore, previous declines could not have been caused by human impact. If the recent decline is outside the historic range of variability, then it could be due to recent climate change and/or increased human activities. Comparison of tree growth in declining and non-declining sites would help identifying which factor plays a role in modifying the growth declines.

## Materials and Methods

### Study Area

Our study sites are located in Hengshan Park (39°40′N, 113°44′S) and Wutaishan Park (38°54′N, 113°35′S), which have an area of 148 ha and 593 ha, respectively. These two parks are important sacred sites of Taoism and Buddhism in northern China since the Han Dynasty about 2000 years ago. Since the incorporation of these sites into park management a few decades ago, there have been an obvious increase in the number of visitors to these parks (Zhang, 2009; Xue, 2013).

According to the observed record at the meteorological station in Wutaishan, mean annual air temperature is 6.8°C, and mean annual total precipitation is 566.8 mm. Located 78 km northward, Hengshan is relatively drier than Wutaishan (Ru and Zhang, 2000; Shangguan, 2001).

**FIGURE 1:**
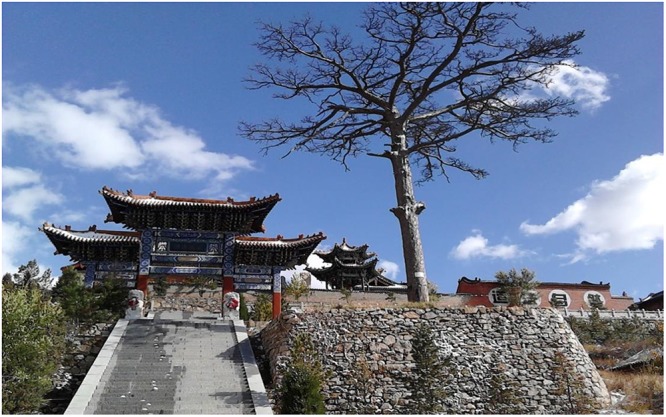
An isolated 350-year old tree (*Pinus tabuliformis* Carr.) in Hengshan Park, Shanxi, China.

The natural forests in the parks are well preserved, with the dominant tree species being Chinese pine (*Pinus tabuliformis* Carr.). Old trees are mostly scattered throughout Hengshan Park, but have a clustered distribution in Wutanshan Park. Recently, some of these old trees have become unhealthy, showing typical symptoms of decline such as crown thinning, foliage discoloration and dieback. Four iconic pine trees in Hengshan Park have died (**Figure 1**).

### Tree-Ring Sampling

At each site, we collected increment cores from the oldest pine trees as judged by stem size, old growth appearance and traditional knowledge of the local people. The elevation of sample collection is 1900–2020 m in Hengshan Park and 1650–1800 m in Wutaishan Park. One core per tree was collected at breast height using a 4.5 mm inner diameter increment borer. In total, we collected 20 samples (including the four dead iconic trees) at Hengshan Park and 36 samples at Wutaishan Park.

In the laboratory, all the cores were air dried, mounted on grooved wooden boards and polished with sandpaper of increasing grit to make the ring boundaries clearly visible (Stokes and Smiley, 1968). Tree-ring widths of each sample were measured to the nearest 0.001 mm in precision using a LINTAB system (@Frank Rinntech Company, Heidelberg, Germany). All samples were cross-dated following dendrochronological techniques so that each ring was assigned to the calendar year of its formation (Fritts, 1976). The quality of cross-dating was checked using program COFECHA (Holmes, 1983). The age-related growth trend in each sample was removed using program ARSTAN in which a negative exponential curve or a cubic smoothing spline with 50% frequency cutoff at two-thirds of the series length was fitted to the measured ring-width series (Cook and Peters, 1981). The resultant tree-ring index (TRI) series of each tree form the database for the analysis of growth trajectories of the sample trees. The tree-ring data are available at http://trl.ibcas.ac.cn.

### Tree-Ring Analysis

We defined a period of growth reduction for an individual tree to be a tree’s growth decline when all three of the following criteria were met: (1) the value of TRI was below 1.0 for at least eight consecutive years, (2) the mean value of TRI during the period was below 0.75, and (3) the value of the minimum TRI was below 0.6 for at least 3 years. These criteria were established on the assumption that the normal growth of trees fluctuates around the mean, and the sustained below average growth together with a severe growth reduction is beyond its natural variation, most likely indicating a growth decline (Cailleret et al., 2016). The exact values for the criteria could be modified to address the severity of the growth declines.

Besides identification of growth declines in individual trees, a forest decline event was recorded if more than half of the trees were declining during the same time. For each period of forest decline that was found, we further classified the declining trees into two types based on the duration of the growth decline. (1) Heavily declining trees, which had periods of growth reduction equal or longer than 20 years, (2) moderately declining trees, which had periods of growth reduction lasting 8 years or more but less than 20 years. We used a Chi-square test to determine if the two sites differed in decline severity. The Chi-square test is constructed from a sum of squared errors and is used to reject the null hypothesis when there is not coherence in data distribution (Flora, 1982).

To identify the influence of climate on tree growth, we calculated the Pearson correlation coefficients between averaged TRI of both non-declining and declining trees (including both heavily and moderately declining trees) and climatic variables. The climatic variables included monthly mean temperature and total monthly precipitation during 1955–2010. We also calculated the correlation coefficients between TRI and Palmer Drought Severity Index (PDSI), a measure of soil moisture conditions that takes into account the influence of both precipitation and temperature (Dai et al., 2004). The PDSI data were extracted from the global data set^1^ for the grid (2.5 × 2.5° latitude and longitude) covering the sampling sites in the period 1955–2010.

## Results

All the samples collected from Hengshan Park and Wutaishan Park were successfully cross-dated. The mean inter-serial correlation, which measures the growth similarity among individual trees, was 0.56 for Hengshan Park and 0.64 for Wutaishan Park, respectively. The average sensitivity of the tree-ring series, a measure of the variability between rings, was 0.22 for Hengshan Park and 0.24 for Wutaishan Park, indicating that these trees were sensitive to environmental changes. The mean breast height age of the sample trees was 256 years old at Hengshan (oldest tree was 356 years old) and 265 years old at Wutaishan (oldest tree was 481 years). Because the number of samples decreased back in time, we selected the interval A.D. 1778–2010 during which both sacred sites had at least 12 sample replications for the analysis of historical growth declines.

**FIGURE 2:**
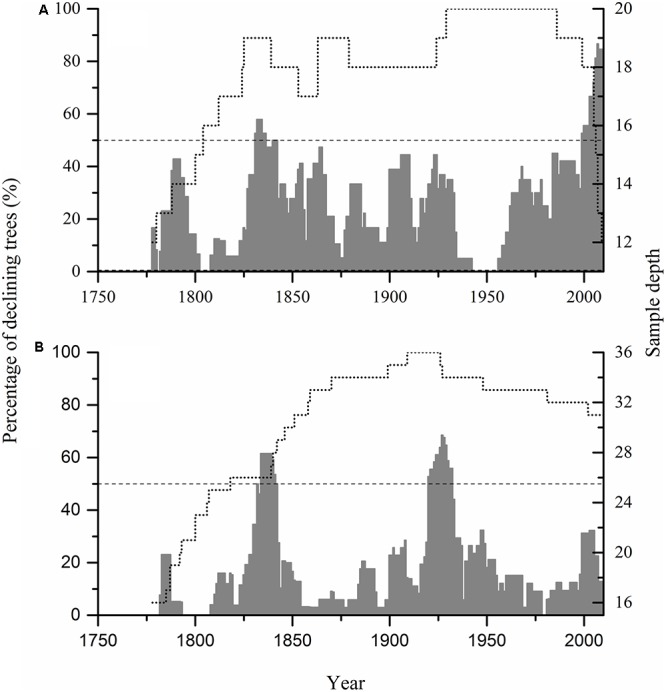
Percentage of growth declining trees during 1778–2010 in Hengshan Park **(A)** and Wutaishan Park **(B)**. Dotted line represents the number of tree-ring samples; dashed line represents the threshold defining forest decline events.

**FIGURE 3:**
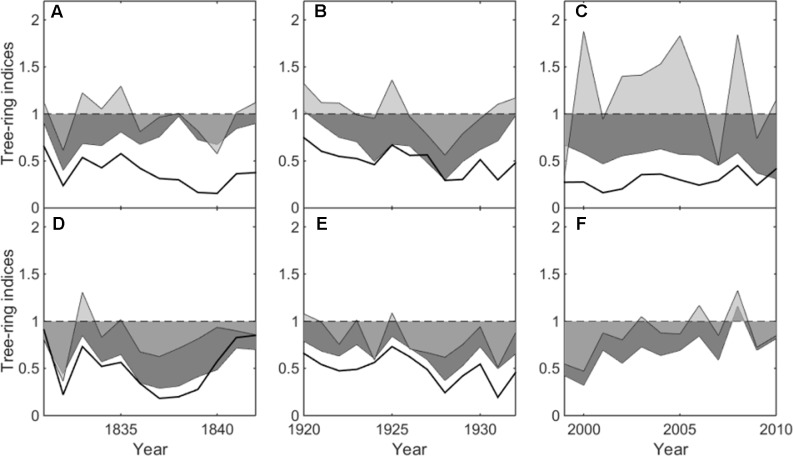
Mean ring-width indices from heavily declining trees (reduced-growth lasting more than 20 years) (black line), moderately declining trees (reduced-growth lasting more than 8 but less than 20 years) (dark grey shadow) and non-declining trees (light grey shadow) in the three growth declining events (1831–1842, 1920–1932 and 1999–2010). **(A–C)** refer to Hengshan Park and **(D–F)** refer to Wutaishan Park.

Examination of the growth trajectory of individual trees showed that trees with periods of growth decline (>8 years in duration) were ubiquitous throughout the past 233 years. However, declines that affected more than 50% of the trees occurred during three events in the interval of analysis (**Figures 2**, **3**).

### The 1830s Decline (1831–1842)

This decline was evident at both sites, affecting as many as 58 and 62% of trees in the most severe year at Hengshan and Wutaishan, respectively. The percentage of trees with heavily reduced growth was 26% at Hengshan but only 8% at Wutaishan.

### The 1920s Decline (1920–1932)

This decline occurred only at Wutaishan Park, affecting up to 67% of the trees in the most severe year. At Hengshan Park, the percentage of declining trees did not reach 50% but was as high as 44%.

### The 2000s Decline (1999–2010)

**FIGURE 4:**
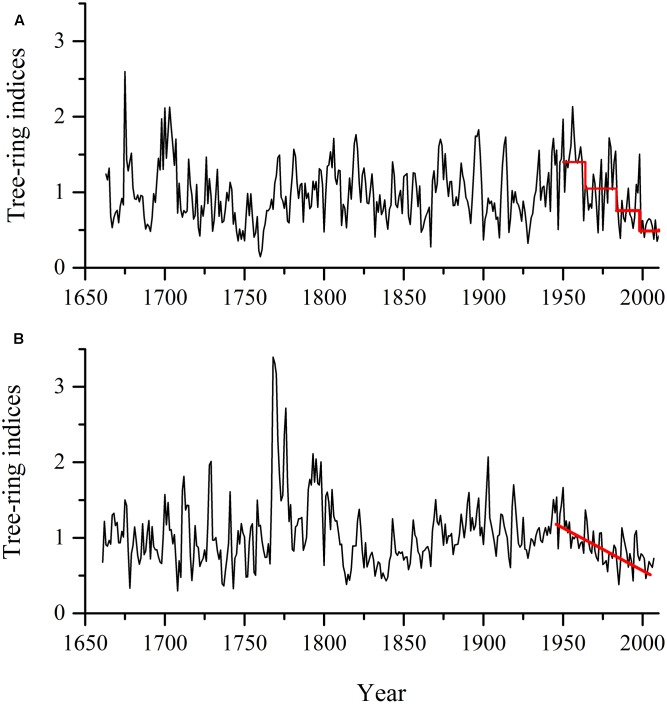
Average radial growth indices in sixteen living *Pinus tabuliformis* Carr. trees **(A)** and in four dead trees **(B)** in Hengshan Park, Shanxi, China.

This decline occurred only at Hengshan Park and was the most severe event in the past 233 years. The number of declining trees showed an increasing trend since the 1960s and was still continuing at the time of sampling. The percentage of declining trees reached 86% and the heavily declining trees 32%. Contrary to the situation at Hengshan Park, the percentage of declining trees in Wutaishan Park was below 30% since the 1960s and none of the trees were heavily declining. The proportion of declining trees was statistically higher at Hengshan than at Wutaishan (χ^2^ = 21.21, *p* < 0.05, **Table 1**). The proportion of non-declining trees was 71% at Wutaishan but 10% at Hengshan. The growth trajectory of the four dead trees in Hengshan Park showed a five-decade growth decreasing trend before death, a much greater decline than in the surviving living trees (**Figure 4**).

**Table 1:** Number and percentage of *Pinus tabuliformis* Carr. trees that sustained three decline episodes at Hengshan and Wutaishan Parks.

Decline period	1831–1842			1920–1932			1999–2010		
Types	H	M	N	H	M	N	H	M	N
Hengshan	5 (26%)	8 (42%)	6 (32%)	5 (25%)	7 (35%)	8 (40%)	6 (32%)	11 (58%)	2 (10%)
Wutaishan	2 (8%)	16 (61%)	8 (31%)	7 (20%)	19 (53%)	10 (27%)	0 (0)	9 (29%)	22 (71%)
χ^2^	3.23			1.66			21.21		
*P*	0.20			0.44			<0.05		

*Trees were sorted in three classes based on the length of the observed decline, H, heavily declines lasting more than 20 years; M, moderately declines lasting more than 8 but less than 20 years. N, non-declining*.

**FIGURE 5:**
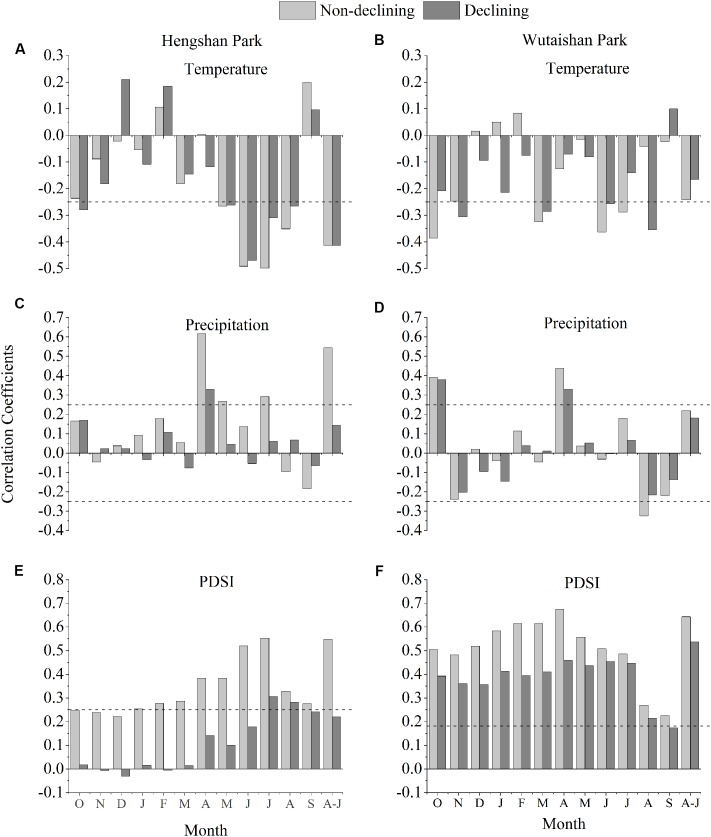
Correlation coefficients between tree-ring chronologies and monthly PDSI in Hengshan Park **(A,C,E)** and Wutaishan Park **(B,D,F)**. The months include October of the prior growth year to September of the current growth year, and April–July. The dotted lines represent level of significance at *p* < 0.05.

Analysis of tree growth-climate relationships showed that TRIs from non-declining trees were correlated with temperature and precipitation in April to July and were significantly and positively correlated with April-July PDSI in both sites (**Figure 5**). This relationship weakens in declining trees in general in recent times especially in Hengshan.

## Discussion

We investigated tree growth declines using a new method in this study. We consider that tree-ring indices fluctuate around their mean when the tree is in normal growth. Trees that show below average growth sustaining for a number of years and at certain level of growth reduction are likely in growth declines. The exact values for the criteria of duration and severity could be modified to reflect the characteristics of the growth declines.

We identified three intervals of forest decline in the study area during the past 223 years. At Wutaishan Park, growth declines occurred in the 1830s and 1920s and were absent in recent years, suggesting that the driving factor for forest declines has remained unchanged in the past 223 years, and climate or natural disturbance is the main factor for the past declines. Although human activities are increasing in recent years at Wutaishan Park, their impacts are somehow reduced or other factors are involved to increase their ecological resistance to disturbance (Thompson et al., 2011).

At Hengshan Park, there was a strong decline of growth in recent decades. The observation that the recent decline is outside the historic range of variability suggests a change in the forces driving the declines. Given that both climate has changed and human activity has increased, it is difficult to distinguish which of the two contributed to the tree-growth decline in the 2000s. In comparison, the trees in Wutaishan Park did not show an intensified growth decline in the recent than the past. To interpret the difference in the decline pattern between Hengshan and Wutaishan, we hypothesize that the resistance of tree growth to external disturbances is forest size dependent. One obvious difference between the two parks is their size, with Wutaishan Park (593 ha) being four times larger than Hengshan Park (148 ha). It is reported that isolated populations in small forest patches have low stability and face high risks of mortality (Newmark, 1998; Jacquemyn et al., 2001; Turner et al., 2007). The role of root systems in maintaining soil stability and water holding capacity might be limited in fragmented forests, thus reducing the ability of trees to obtain enough water (Newmark, 1998).

Climate-warming induced drought has been evidenced to be a major natural force causing sustained growth decline of trees and forests in many regions of the northern hemisphere during recent decades (Anderegg and Field, 2012; Chen et al., 2017a,b), especially in arid and semi-arid regions. Growth decline of *P. tabuliformis* and *P. bungeana* forests induced by climatic drought in the southeastern Shanxi province was also reported (Zhang et al., 2017). In fact, the climate in Hengshan park is relatively drier in April–July than Wutaishan (Ru and Zhang, 2000; Shangguan, 2001), although the two parks are only about 78 km apart. The tree growth-climate relationship analysis demonstrated that narrow tree rings are associated with dry condition in April–July (**Figure 3**).

The much stronger growth decline in recent decades than the past at Hengshan Park also suggests a linkage with the increasing use of the site by people, if the climate change is not the only factor causing such highly intensified decline. The average amount of visitors is about three million per year to Wutaishan Park and seven hundred thousand to Hengshan Park (Zhang, 2009; Xue, 2013). The ground pavement, construction and urbanization in the scenic area were started only a few decades ago (Xue, 2013; Zhang et al., 2013). Ground paving during park construction and soil compaction caused by tourist disturbance can affect tree growth by reducing storage of moisture and capillary volume of the soil (Thompson et al., 2011; Hauru et al., 2012; Sikorski et al., 2013). Fragmentation of forests caused by human activities is also harmful for the growth of old trees because it increases the risk of old trees’ being exposed to intense sunlight and evaporation (Laurance et al., 2000). The growth of non-declining trees was positively correlated to April–July moisture condition, but such correlation disappeared in the growth of declining trees (**Figure 3**), suggesting that the climate-growth response in declining trees has been altered by human activity (Sikorski et al., 2013; Seddon et al., 2016). The absence of intensified decline in the recent decades at Wutaishan may simply be the result of a more diffuse impact of the visitors over a large area.

Another possibility of the reduced tree resistance at Hengshan Park might be related to aging effect. Generally, old trees have lower vigor than young trees. It is reported that tree aging plays a role in controlling tree mortality in boreal forests (Luo and Chen, 2011). The rates of photosynthesis reduce with their age and growth in size (Lugo and Scatena, 1996), and this aging effect is also related to changes in completion of using resources (Dan et al., 2002). In our study, the aging effect might be a factor contributing to the death of the four old trees at Hengshan Park. Because the aim of this study was to detect growth declines in history, our sampling strategy was to select trees as old as possible for drilling increment cores. The shortage of samples from different age classes prevented us from testing aging effect on tree decline. However, in our sampled trees, the frequency of growth decline events did not increase with age in the past 223 years, suggesting that, before the recent decline event, these old trees were healthy to minimize the effect of aging on growth declines. If the external disturbances increase their intensity in future, the aging effect would probably emerge and these old trees would face higher risks to declines than young trees.

The number of declining trees in the 1830s and the 1920s at the two parks had no significant difference (**Table 1**), and the growth trajectories in trees of different decline duration showed similar pattern in these two events (**Figure 3**). Such similarity suggests that the influence of external factors on forest growth at these two parks were not too severe to exceed the capacity of ecological resistance of the forest size. Human activity might not be a factor driving these earlier growth declines, neither climate warming. Other natural disturbances, such as insect outbreaks and storms, although not observed in the recent decline at both Wutaishan and Hengshan Parks, might occur in tree decline events in the 1830s and 1920s.

We found difference in growth response of individual trees to environmental stresses. In a forest decline event, although quite a number of trees declined, other trees in the same forest did not decline (**Figures 2**, **4**). Clearly individual trees had different resistance to declines and also there were differences in micro-habitats of individual trees (McNulty et al., 2014; Galván et al., 2015; Vindenes and Langangen, 2015). We observed that the trees having a period of decline were more ubiquitous at Hengshan Park than at Wutaishan Park during the past 200 years (**Figure 2**). This observation was likely a result of difference in the size of the parks and in the climate conditions.

## Conclusion

In summary, our tree-ring data reveals that forests in the sacred sites of Hengshan and Wutaishan Parks experienced three events of growth declines in the past 200 years, suggesting that these forests, although benefit from local protection, were still susceptible to climate change and natural disturbances. Relative to Wutaishan Park, trees in Hengshan Park had more ubiquitous growth declines in the past and a stronger decline in the recent decades. In this context, the Hengshan forest is a much smaller fragment than Wutaishan and can be more sensitive to stress factors, particularly in face of combined effects of climate change and human activity at present. In conservation of old growth forests in small and harsh-conditioned parks, managers should consider reducing fragmentation of old forests and restoring natural habitat for old trees. Identification of the pattern of past declines in tree growth is a useful approach to assess ecological resilience to natural disturbances and human activities.

## Author Contributions

Q-BZ initiated the study, organized field work, supervised the process of data analysis, and manuscript writing. YL conducted the data analysis, and wrote the manuscript.

## Conflict of Interest Statement

The authors declare that the research was conducted in the absence of any commercial or financial relationships that could be construed as a potential conflict of interest.

## Footnotes

1 http://climexp.knmi.nl/start.cgi
